# Design of a Combined Modular and 3D-Printed Falling Film Solution Layer Crystallizer for Intermediate Purification in Continuous Production of Pharmaceuticals

**DOI:** 10.1021/acs.iecr.1c00988

**Published:** 2021-07-12

**Authors:** Rafael Lopez-Rodriguez, Matthew J. Harding, Geoff Gibson, Kevin P. Girard, Steven Ferguson

**Affiliations:** † School of Chemical and Bioprocess Engineering, University College Dublin, Belfield, Dublin 4, Ireland; ‡ SSPC, The SFI Research Centre for Pharmaceuticals, School of Chemical and Bioprocess Engineering, University College Dublin, Belfield, Dublin 4, Ireland; § I-Form, The SFI Research Centre for Advanced Manufacturing, School of Chemical and Bioprocess Engineering, University College Dublin, Belfield, Dublin 4, Ireland; ∥ Pfizer Ireland Pharmaceuticals, Ringaskiddy, Ireland; ⊥ Pfizer Inc. Chemical R&D, Groton, Connecticut 06340, United States; # National Institute for Bioprocess Research and Training, 24 Foster’s Avenue, Belfield, Blackrock, Co. Dublin A94 X099, Ireland

## Abstract

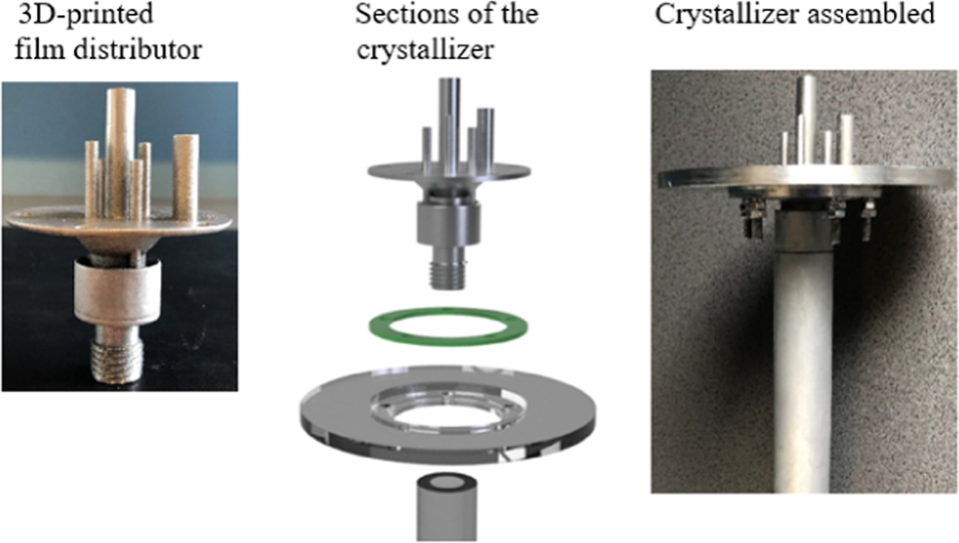

A highly scalable combined modular and 3D-printed falling film crystallization device is developed and demonstrated herein; the device uses a small, complex, printed overflow-based film distribution part that ensures formation of a well-distributed heated liquid film around a modular, tubular residence time/crystallizer section, enabling extended residence times to be achieved. A model API (ibuprofen) and impurity (ibuprofen ethyl ester) were used as a test system in the evaluation of the novel crystallizer design. The proposed crystallizer was run using three operational configurations: batch, cyclical batch, and continuous feed, all with intermittent removal of product. Results were suitable for intermediate purification requirements, and stable operation was demonstrated over multiple cycles, indicating that this approach should be compatible with parallel semicontinuous operation for intermediate purification and solvent swap applications in the manufacture of drugs.

## Introduction

Continuous reactions and processes have been standard practice for decades in the commodity chemicals sector.^1^ The last 15 years have seen significant interest and development in the synergistic application of continuous reaction technologies, novel chemistries, and often overlooked chemical processing and separation technologies for the synthesis of small volume, but more complex chemical products. This has produced advances in R&D and chemical processing methodologies for many chemical products, for example, nanoparticles (inorganic/organic),^2,3^ polymers,^4−6^ proteins,^7^ peptides,^8^ oligonucleotides,^9,10^ and pharmaceuticals.^11−14^

Continuous manufacturing of modern pharmaceuticals typically requires a significant number of synthetic transformations with high product purity requirements. It has enabled novel chemistries not compatible with batch-tank-based scale-up to be investigated, for example, highly exothermic reactions,^15^ generation of hazardous intermediates,^16^ as well as photochemical^17^ and electrochemical transformations.^18^ The use of continuous manufacturing results in a step change in process intensity with orders of magnitude reduction equipment scale and improved safety, and is more amenable to process control and automation. Furthermore, it may be an enabler for the development of new approaches such as integrated co-processing of active pharmaceutical ingredient (API) and drug product,^19^ real-time release, shortened supply chains in pharmaceuticals, and modularization of supply.^20^

Crystallization is a key operation in pharmaceutical production, and a number of continuous crystallization platforms have been demonstrated to be viable, with sufficiently robust operation, while being capable of commercial levels of supply.^21^ Continuous stirred tank reactors (CSTRs), mixed suspension mixed product removal (MSMPR) crystallizers, and tubular and oscillatory flow crystallizers have been demonstrated to be able to monitor, characterize, model, and control all important crystal product attributes for pharmaceuticals such as crystal size distribution (CSD),^2,13,14,22−25^ morphology,^13,14,22^ chemical purity,^11,12,26^ yield,^11,12,26,27^ chirality,^28^ or polymorphic purity.^28−32^

Despite significant development in the field of continuous crystallization for pharmaceutical applications over the past decade it remains a relatively challenging operation to design and operate for the purpose of intermediate purification. Intermediated purification via continuous crystallization has the requirement to handle supersaturated streams with long residence times,^33^ transfer slurries, filter,^34^ in some cases convey wet filter cake, wash, and dissolve for further processing. As such, conventional continuous crystallizers cannot easily fulfill the workhorse role for intermediate purification and solvent swapping as batch crystallization in pharmaceutical synthesis. Batch synthetic routes often define “steps” by the intermediate isolations, which provide operational flexibility and offer significant intermediate purification, allowing each step to start with ideal conditions. It is therefore potentially desirable to maintain the flexibility of batch crystallization in telescoped, integrated continuous routes to meet the high-purity specifications for APIs. Solution-layer crystallization is an option to retain some of the power of intermediate suspension crystallization without the need for significant intermediate isolation.

Falling film layer crystallizers are established for use in melt crystallization or in progressive freeze crystallization.^35^ Crystallization occurs on cooled surfaces forming a crystal layer, which removes water/ice crystals in freeze crystallization or produces the desired product in melt crystallization. However, high temperatures needed in melt crystallization are not usually suitable for APIs and many pharmaceutical intermediates, due to the need for compounds to spend significant residence times above their melting point.^33^ Falling film solution layer crystallization enables isolation free crystallization, eliminating the handling of slurries/solids and obviating the need for filtration,^33,34^ all while using supersaturated solutions below melting or degradation temperatures. The product is recovered by crystallization on a cold finger with high purity, while the impurities preferentially partition into the mother liquor. A key characteristic is to combine the steps of crystallization, isolation, drying, and redissolution into a simple piece of equipment with no moving parts, with no slurry or solids handling, and with no isolation needed.

Smaller process equipment developed for continuous manufacturing/crystallization has coincided with technological developments in additive manufacturing, also known as 3D printing. 3D printing can enable the customization of equipment for specific process requirements with high precision and complex geometries within parts that would be prohibitively expensive with traditional fabrication methodologies.^36−39^ Protein crystallization of lysozyme was conducted recently in an airlift crystallizer constructed from sections printed by stereolithography (SLA).^40^ Another example is the CSTR platform, printed in both resin via SLA and 316L stainless steel by selective laser melting (SLM), for the production of functionalized silica particles with size below 100 nm.^2^ However, there are limitations in material choice, especially in advanced alloys for acid-compatible metal printing. Many of the polymers available for 3D-printing applications also exhibit poor chemical compatibility with typical reagents and solvents used in pharmaceutical flow synthesis. The ability to print complex internal structures and long channels needed for flow chemical applications in metals can require major postprocessing steps to remove powder.^41^ Fused filament fabrication/fused deposition modeling (FFF/FDM) printers avoid the need to clear powders but introduce geometry constraints relating to overhanging faces and limitations in terms of chemical compatibility.^42^ Recently 3D printing of poly(etheretherketone) (PEEK) reactors compatible with pressures of up to 60 bar has been demonstrated, which extends the utility of FFF/FDM-printed parts into higher chemical-, pressure-, and temperature-resistance-requiring applications.^42^

Above all, the scale of 3D-printed parts with desirable characteristics for chemical processing remains a limitation to their uptake. Furthermore, as the rate of reaction decreases, residence time requirements increase and the advantage of highly engineered heat and mass transfer improving geometries also recedes, reducing the added utility of printed over modular parts with more conventional geometries. In this work, we outline a design approach that aims to keep the strengths of additive manufactured parts, but combines them with modular, off-the-shelf parts to enable increased process scale, facilitate flow processes, and allow direct scale-up for continuous supply of medicines. To this end, a new falling film solution crystallizer design was developed and fabricated incorporating modular, 3D-printed parts coupled to traditionally machined parts for continuous production of pharmaceuticals. The crystallizer was characterized under several operation modes: parallel batch, cyclical batch, and continuous feed with intermittent removal of product.

## Materials and Methods

### Materials

Ibuprofen (purity ≥ 99.5%) was bought from Kemprotec Limited U.K. and used as received. Ethanol (purity ≥ 99.8%) was bought from Fisher Scientific U.K. and used as received. Deionized water (18.2 MΩ) was used along with ethanol to prepare the different solutions for the experiments with ibuprofen. Ibuprofen ethyl ester was used as impurity and was synthesized from ibuprofen via Fischer–Speier esterification and isolated (details in the Supporting Information). The initial feed solution consisted of 2.0 g ibuprofen/g solvent. The solvent ratio was 3.99:1 ethanol/deionized water by weight. The target impurity in the initial feed was 4.5% by relative peak area with respect to ibuprofen. As the impurity was produced, some variations were found in the feed solution. An HPLC method was developed to analyze the purity of the ibuprofen/ibuprofen ethyl ester mixture, which is described in more detail below. The initial feed solution was kept in a water bath at 62 °C and mixed with a magnetic stirrer until the ibuprofen was dissolved.

### HPLC Analysis

Purity analysis was performed using a 1100 series HPLC (Agilent Technologies, Santa Clara) using a C18 reversed-phase column (Hypersil BDS, 5 μm PS, 4.6 × 150 mm^2^, Thermo Fisher, Waltham). The mobile phase was isocratic with 60:40 CH_3_CN/H_2_O with 0.05% v/v TFA added. The ibuprofen and the ibuprofen ethyl ester were analyzed using a flow rate of 1.2 mL/min, injection volume of 5.0 μL, column temperature of 30.0 °C, detection wavelength of 220 nm (16 BW, 360 ref.), and running time of 14 min.

### Gravimetric Analysis

Samples were collected during the experiments from the feed, final product, and wash solution vessels. Ibuprofen, ethanol, water, and ibuprofen ethyl ester were weighed at the beginning of the experiment to prepare the initial feed solution. Gravimetric analysis was conducted to determine the yield of the process. Crystallizing dishes were weighed at the beginning of the experiment using an analytical balance (Fisherbrand, readability 0.0001 g, from Fisher Scientific). The sample (250 μL) was added and weighed again. The crystallizing dish was placed in a vacuum oven at 40 °C overnight to remove the solvents. Crystallizing dishes were weighed after at least 15 h in the oven, each sample was analyzed in triplicate, and the average is reported.

### Cooling Batch Suspension Crystallization

A batch suspension crystallization experiment was conducted for comparison using a 100 mL vessel in an EasyMax 102 (Mettler Toledo, U.K.) with overhead stirring at 350 rpm. A steel pitched blade impeller with 45° inclined blades and 38 mm diameter was used. An initial concentration of 1.0 g ibuprofen/g solvent and a solvent ratio of 3.99:1 ethanol/deionized water was used. The solution was preheated to 50 °C to dissolve the ibuprofen. A lower concentration was used in this batch experiment than during the layer crystallization to enable agitation throughout the experiment. The feed solution (100 mL, 92.01 g) was used, which had an initial ibuprofen purity of 95.52% by relative peak area (impurity 4.48% by relative peak area). A cooling crystallization was conducted as follows: cooling down from 50 to 35 °C at 1 °C/min, constant temperature at 35 °C for 30 min, cooling down to 20 °C at 1 °C/min, constant temperature at 20 °C for 30 min, cooling down to 8 °C at 1 °C/min, and constant temperature at 8 °C for 60 min. Total time for the experiment was 162 min. The final temperature was the same as in the tube side in the falling film crystallizer (FFC). At the end of the experiment, the crystals produced were vacuum-filtered and weighed. A washing step was then conducted using 40 mL of ethanol at 8 °C, vacuum-filtered, and dried to calculate the yield.

## Falling Film Crystallization Experimental Setup

Falling film crystallization experiments were conducted utilizing a similar overall experimental configuration to previous demonstrations in the literature^33^ but using the novel combined modular 3D-printed equipment.

**Figure 1:**
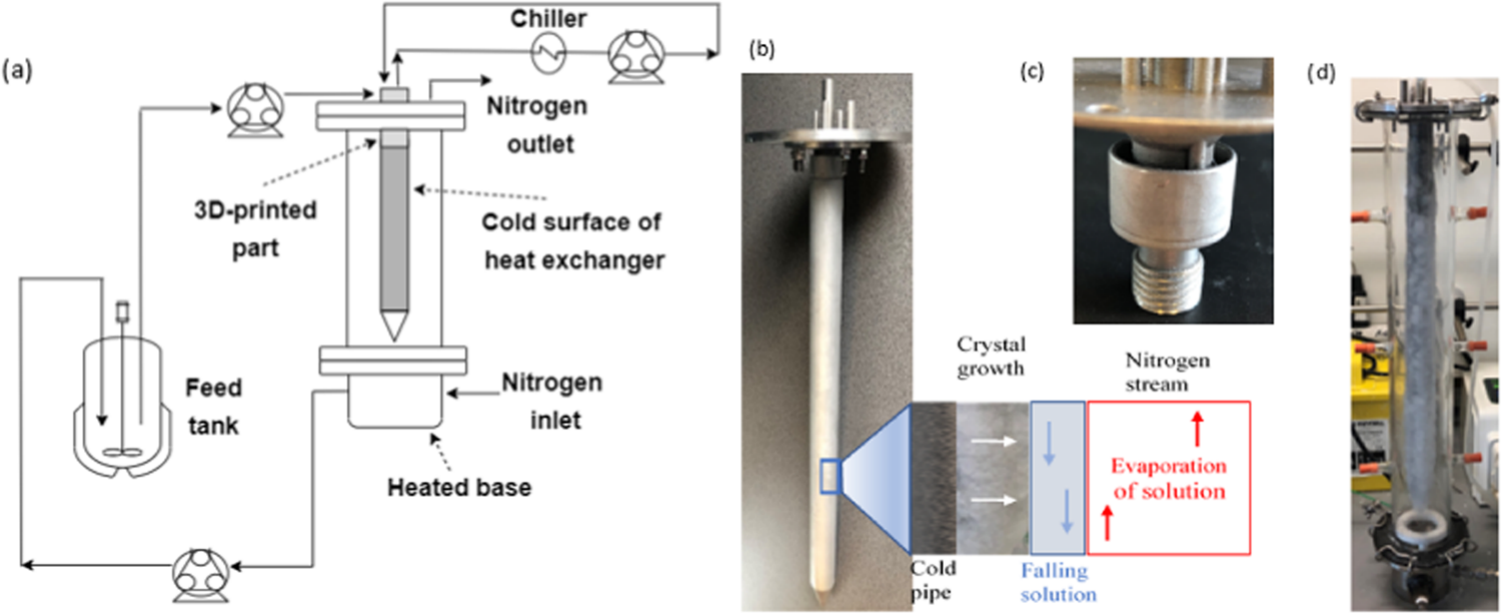
(a) Process diagram of the falling film solution layer crystallizer, (b) details of the crystallization process on the cold surface of the heat exchanger, (c) details of the open reservoir to form the film around the heat exchanger, and (d) FFC with the crystallized product.

The FFC utilized a 1.0 L Duran bottle as the feed tank, which contained a supersaturated solution of a desired ibuprofen and impurities dissolved in the solvent, which was heated using a hot plate (Wisd, MSH-20D-SET with PT100 temperature probe) and water bath at 62 °C. The feed solution was pumped using a peristaltic pump (Watson Marlow, 101U/R, 0.4–53 mL/min) to the FFC at 10.50 mL/min, where it was distributed by a 3D-printed film distributor, with an internal, heated reservoir to prevent crystallization in the distributor section. The falling film used in the crystallization operation was generated via overflow from a cylindrical trough by pumping the feed flow through the 3D-printed film distributor (Figure 1c). This film the flows downward under gravity onto a cooled tubular pipe section which was attached using ISO-parallel male printed threads on the printed distributor part, which screwed into a 1″ (25.4 mm) OD stainless steel pipe tapped M16 ISO-parallel threads. Figure 1 presents the process diagram of the FFC and the details of the crystallization process.

**Figure 2:**
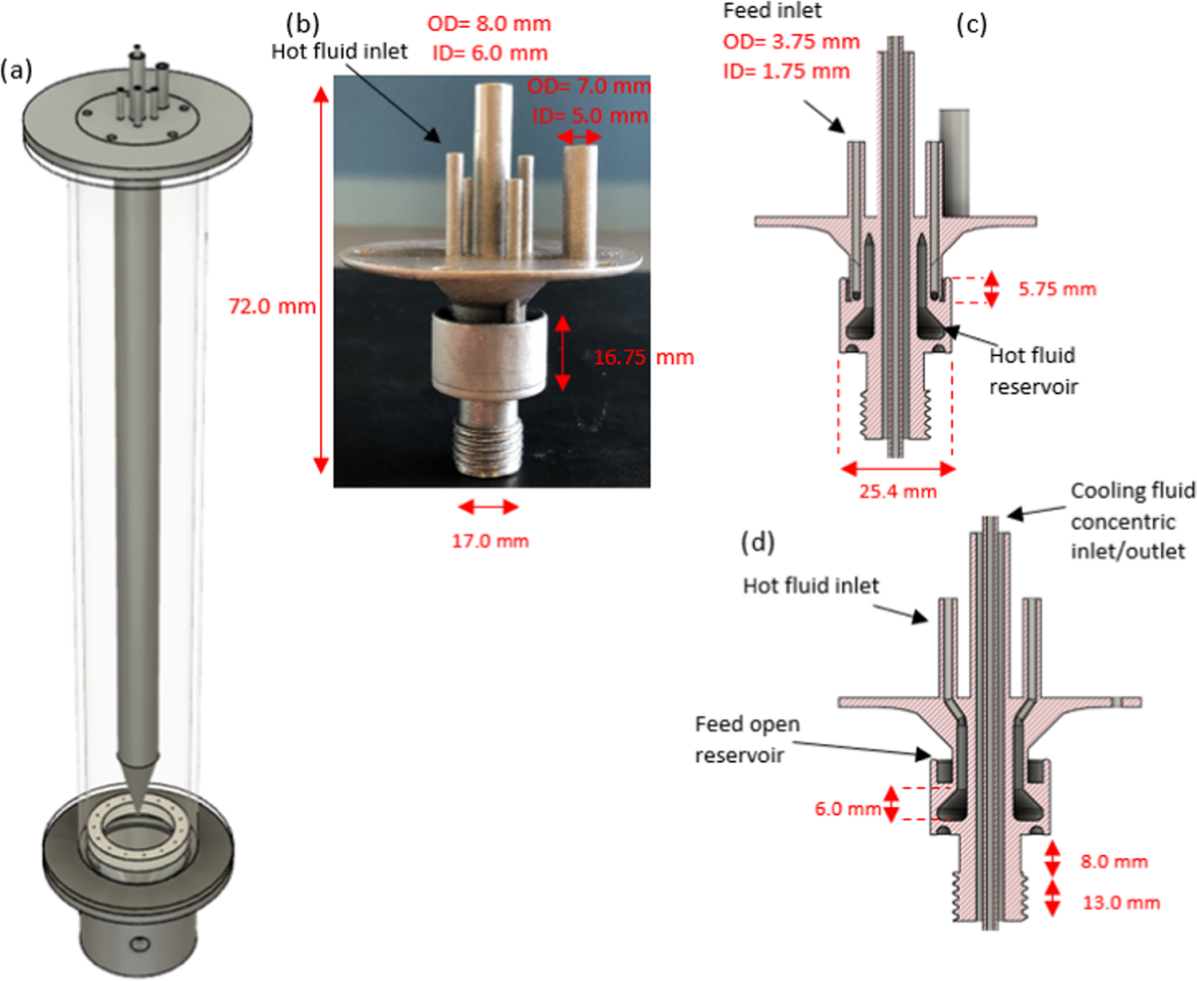
(a) Design of the falling film solution layer crystallizer with 3D-printed parts, (b) film distributor 3D-printed, (c) cross-sectional view over the *X*, axis, and (d) cross-sectional view over the *Z* axis of the film distributor.

The tubular section was cooled via recirculation of fluid using an external pumped heater/chiller circulator (Julabo, F25-ME refrigerated/heater circulator) to generate supersaturation to drive crystallization of the API. The crystallizer had a single-contact-point cold finger style heat exchanger using a tube-in-tube inlet/outlet. To achieve this, a 1/8″ (3.175 mm) polypropylene (PE) tube was fed down through the central pipe (outer diameter = 8.0 mm) in the 3D-printed distributor and tubular residence time section just above the conical bung (detailed figures in the Supporting Information). Figure 2c,d presents the cross-sectional views of the 3D-printed film distributor with the inserted 1/8″ pipe. Coolant was introduced at ∼24 mm above the bottom of the tubular heat exchanger and flowed out at the top of the device where the fluid was recirculated back into the chiller using Swagelok connectors.

A nitrogen stream was injected from the bottom through a sparger, with flow rate controlled by a 0.6–5 L variable-area flow meter (CT Platon, NG series GTF2BHD). Nitrogen gradually removes solvent via evaporation, generating supersaturation in addition to that generated by cooling. All of the experiments were conducted using a nitrogen flow rate of 4.0 L/min. The solvents and solutes that are not crystallized in a pass through the crystallizer are collected in the conical base of the equipment, which was kept at 62 °C to prevent crystallization of the product and blockages. The excess fluid was pumped back into the feed tank using a peristaltic pump (PLP-380, behr Labor Technik, Düsseldorf, Germany). All process lines were kept hot using tube-in-tube configurations and circulation of hot water. The internal pipe was 1/8″ OD × 0.028″ (0.71 mm) wall stainless steel tubing (Swagelok, SS-T2-S-028-6ME) to pump the feed solution, and the external pipe was 3/8″ (9.525 mm) OD × 0.035″ (0.89 mm) wall stainless steel tubing (Swagelok, SS-T6-S-035-6ME) for hot water, which was circulated using a gear pump (Ismatec, MCP-Z, 1–7020 mL/min, Germany), and the temperature was controlled using the probe from the hot plate.

## Design and Fabrication of Combined Modular and 3D-Printed Falling Film Solution Layer Crystallizer

A new falling film solution crystallizer (FFC) design was developed and fabricated incorporating 3D-printed parts with modular parts aimed at continuous production of pharmaceuticals. Figure 2a shows an overall render of the modular and 3D-printed FFC, with Figure 2b showing the 3D-printed distributor and Figure 2c,d presenting cross section over the *X* and *Z* axes, the dimensions and internal features. The crystallizer was divided into three sections: a 3D-printed film distributor (heated), a cooled heat exchanger over which the film flows under gravity and where crystallization occurs, and a conical bung from which solution fell and was collected for recycle to the feed tank and fed back to the distributor. These segments comprise the active process side components in the crystallization process that can be assembled by combining the 3D-printed distributor with off-the-shelf, modular components, by simply screwing them together. To enable this, M16x1.5 ISO-parallel threads were printed into the distributor, which could be screwed into the matching M16 ISO-parallel threads tapped within the tubular section.

The crystallizer can be housed by hanging from the flanged section of the 3D-printed distributor inside a simple column with flanged fittings and clamps used to seal the glass sections to a plate to the top and a heated conical well from which solution was maintained in above the saturation temperature and recirculated to the feed tank. The column used here was not the primary objective of the design in this study. The column height was sized to fit within a standard laboratory fume hood and be operable by a single researcher and was 665 mm with 120 mm outer diameter. Further details of its construction and parts used can be found in the Supporting Information. Due to the modular nature of the design, the cold surface area of the crystallizer or residence time on the film can be increased by simply screwing in a longer tubular section and numbered up by hanging multiple units housed in a larger glass column or vessel. For this reason, design of a single point of contact heat layer crystallizer was a key design objective in enabling numbering up of units.

The use of a 3D-printed film distributor enabled a complex and novel distributor geometry to be developed to meet a number of design criteria for the overall device that would be challenging to meet with traditional fabrication methodologies. These characteristics include homogeneous external film distribution, novel independent dual heat exchanger configuration, modularity, scalability, and securing a design that could be printed in the direct metal printing/selective laser melting (DMP/SLM) technique used for the fabrication in 316L stainless steel. A detailed description of the geometry of the 3D-printed device and the overall column is provided in the Supporting Information, with additional schematic diagrams provided.

- Homogeneous distribution of the feed solution: It is a prerequisite for a successful film-based operation. The film flows on the outside of the device to avoid blockages as per Figure 2b–d. Furthermore, this configuration enables visual observation of the progress of the crystallization and during cleaning, which is often useful in GMP production for the manufacture of drugs. In this device, a uniform external film distribution is achieved by an overflow design from a trough as per Figure 2b,c. This design negates the need to have a narrow and uniformly controlled gap, often 1 mm,^33^ for film formation between components, and so should be resistant to blockage. The distributor section arches out to form a flange at the top of the device so that simply hanging the device from a leveled plate in a single column or numbered up in a larger column is sufficient for successful distribution.
- Novel independent dual heat exchanger: The ability to print complex internal structures gives the ability to print an internal reservoir for heated fluid to provide local heating in the distributor. This is accomplished through printed 3.75 mm OD tubes extending from the device. The temperature of the distributor can be kept above the solution’s saturation temperature and prevent crystallization in the trough and around the distributor section, which could inhibit the ability to form a uniform film and potentially block the device. In addition, an 8 mm OD pipe with a 6 mm ID opening was maintained through the centerline of the device. A (1/8″ PE) coolant inlet tube was run down the center of the device through the distributor to introduce coolant flow just above the connection for the conical bung, which seals the coolant on the inside of the device (Figure 2c,d). The coolant then flows up, counter current to falling film, and out through the annular gap between the concentric tubes in the device (additional figures in the Supporting Information). Numbering up could be achieved by simply hanging multiple devices inside a single column or vessel.
- Modularity and scalability: As outlined, the design of the 3D-printed distributor makes numbering up of units a relatively trivial task within a standard column or vessel. The 3D-printed threads as part of the distributor enable the increment of the cold area of the crystallizer using longer pipe sections. It also had space for a Viton O-ring (OD 19 mm, 2.5 mm cross section) to prevent leaks from the feed fluid to the cooling jacket or vice versa. A parallel thread was used to ensure that the device can be screwed all the way, preventing significant discontinuity between the segments disturbing film flow. This approach enables the design to be modular, as standard parts such as 1″ pipe can be tapped with the corresponding female fitting and connected directly to the distributor. Thus, the length and hence the productive surface area of the crystallizer can be increased simply by selection of modular parts. The conical bung in the example was machined; however, it could be as easily replaced with a printed equivalent or off-the-shelf flat-ended screw in plug if desired. Wider pipe and hence overall device dimensions could be targeted, if desired by scaling this design proportionally, in addition to provision of additional length or numbering up of units.
- Printability: Finally, the design must be compatible with the 3D-printing equipment being utilized. Powder bed selective laser melting (SLM) of 316L stainless steel was used, and the details are presented in the Supporting Information.

## Results and Discussion

In layer crystallization operations, such as the solution layer crystallization used here, there is a buildup of solid material, that must be periodically removed, and hence multicolumn parallelized operation or buffering capacity around the operation is required for use in continuous manufacturing. This investigation is conducted with a single column to demonstrate the stable operability of the approach, but it is anticipated that parallelization may be a more appropriate strategy for deployment, although with sufficient buffering capacity, a single unit could be utilized.

Unlike standard batch suspension crystallization, solution layer crystallization does not have an obvious endpoint. Where evaporation is incorporated, the solution could be brought to complete dryness without blockage, resulting in no purification but quantitative yield. As such in industrial operation, the choice of endpoint should be selected based on an optimum trade-off between yield, purity, and productivity. Per cycle yield can be increased, but with an associated increase in mother liquor concentration of impurities. For systems with structurally similar impurities, this can increase the incorporation of impurities in the product crystal lattice, based on the partition coefficient for the system as well as physical incorporation within the advancing layer. In addition, a thick crystal layer can increase the heat transfer resistance between the falling film and the heat exchanger, decreasing the productivity of the equipment.^33^ As such, an optimum between these factors must be found in the design of a purification step in solution layer crystallization. Details of the operation modes are presented in the Supporting Information. In all cases, the product was dissolved in ethanol to have a slurry-free product, which can be used directly in downstream operations.

## Single Batch Mode: Complete Product Layer Dissolution in Each Cycle

**Figure 3:**
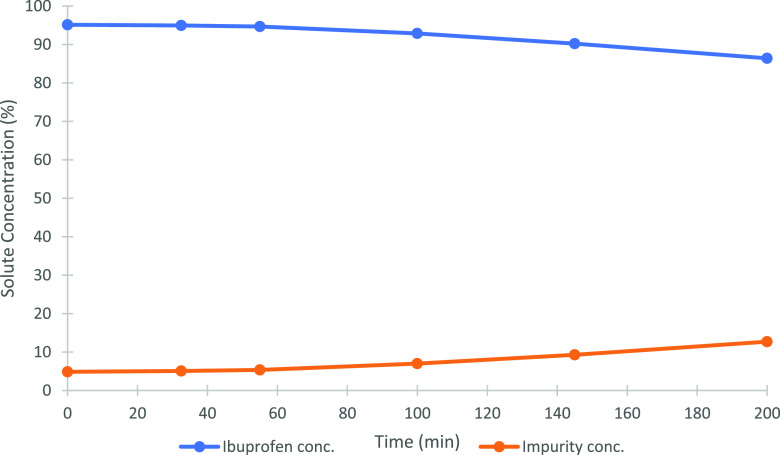
Percentage purity profiles of ibuprofen and impurity in the mother liquor for the single-batch experiment (by relative HPLC peak area).

In this case, the feed solution was created using 218.37 g of ibuprofen, 10.92 g of ibuprofen ethyl ester, 87.30 g of ethanol, and 21.89 g of deionized water. The resulting feed solution was ∼375 mL when the ibuprofen was dissolved. The initial ibuprofen purity in the feed solution was 95.14% by relative peak area (impurity was 4.86% by relative peak area). The flow rate was 10.5 mL/min for feed solution, washing step, and dissolution. The feed solution was recirculated for 200 min, while the ibuprofen was crystallized on the surface of the FFC. Figure 3 and Table 1 show the results for an example single-batch experiment. The relative peak area of the impurity (ibuprofen ethyl ester) compared to ibuprofen in the mother liquor increased during the single-batch experiment, as would be expected due to the preferential partitioning of product (ibuprofen) into the solid phase and the removal of solvent from the mother liquor via evaporation. An increase in the rate of reduction of ibuprofen concentration and increase of the impurity concentration in the mother liquor could be observed after approximately 100 min. This time corresponded visually to the point at which the cooling jacket had been covered with the ibuprofen crystals, providing the full surface for crystal growth, rather than a mixture of growth and heterogeneous nucleation to deposit material on the surface of the device. The supersaturation in the mother liquor was maintained by the evaporation of the solvent.

**Table 1:** Results for the Experiments in the FFC

experiment	batch	cyclical batch				continuous feeding			
washing solvent	100 v% EtOH	100 v% EtOH				100 v% EtOH			
feeding time (min)	200	335				335			
yield ibuprofen (%)	73.15 ± 0.08	76.02 ± 0.52				67.78 ± 0.36			
productivity (g ibuprofen produced/minute of feed)	0.767	0.946				0.90			
		cycle number				cycle number			
		1	2	3	4	1	2	3	4
initial ibuprofen purity (% by relative peak area)	95.14	95.33	95.53						
ibuprofen purity in the redissolved product (% by relative peak area)	98.18	97.90	98.16	98.05	98.29	97.53	97.54	97.05	97.54

At 200 min of operation, the impurity was 12.69% by relative peak area. At this point, the volume left in the feed bottle/tank, reached 100 mL. Lower volumes of mother liquor usually resulted in a purity of the ibuprofen product below 98%. In principle, the crystal layer can run until the system runs dry with no limit on solids loading, unlike agitated batch suspension crystallizations, where 20–33% solids would be considered optimal. Lower solids loading typically with lower yields is also expected in single-pass continuous suspension crystallization, such as MSMPRs, due to their need to operate away from equilibrium.^26^

The ibuprofen purity in the dissolved product was 98.18% by the relative peak area. These results indicated that the FFC successfully removed part of the impurity from 4.86 to 1.82% by the relative peak area. The productivity was defined as
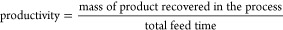
1The yield of the process without secondary washing steps was 77.82% ± 0.09, and the yield after the washing step with 40 mL of 100 v% ethanol was 73.15% ± 0.08, which indicated that part of the product was lost during this step. The productivity was 0.767 g/min. If two crystallizer units were to be used in parallel, one unit can be used to crystallize the product, while the second unit could be used to dissolve it in fresh solvent (semibatch operation mode). In this case, it could be possible to achieve 1.105 kg of product/day using two parallel crystallizers. Further scale-up via numbering up could be possible to achieve 1–10 kg/day. The final purity of the ibuprofen layer can be increased by secondary operations such as washing, for example, 99.39% by the relative peak area in the crystals produced, with a more significant ethanol wash of the layer at the expense of product yield, providing a further opportunity for process optimization.

## Cyclical Batch Operation: Partial Product Layer Dissolution in Each Cycle

To improve productivity in the FFC, cyclical batch experiments were developed using four growth cycles, the initial feed was twice the mass of the single batch solution. In Figure 3, the period of relatively low productivity until 55 min can be seen in the slow change in the concentration of ibuprofen and impurity in the mother liquor, where initial nucleation, nucleation, and growth were developed to cover the surface of the cooling jacket. Description of the cyclical batch operation is presented in the Supporting Information. The first cycle was conducted as per single-batch operation experiments for 170 min; the resulting crystal layer was only partially redissolved using 200 mL of ethanol for 30 min, leaving enough crystal layer covering the cooling jacket. For cycles 2–4, feed was added on top of the remaining crystal layer for 55 min, washed using 40 mL of ethanol at 62 °C for 2 min, and underwent partial dissolution with 200 mL of ethanol for 30 min. At the end of cycle 4, the ibuprofen was fully dissolved using 350 mL of ethanol at 62 °C.

**Figure 4:**
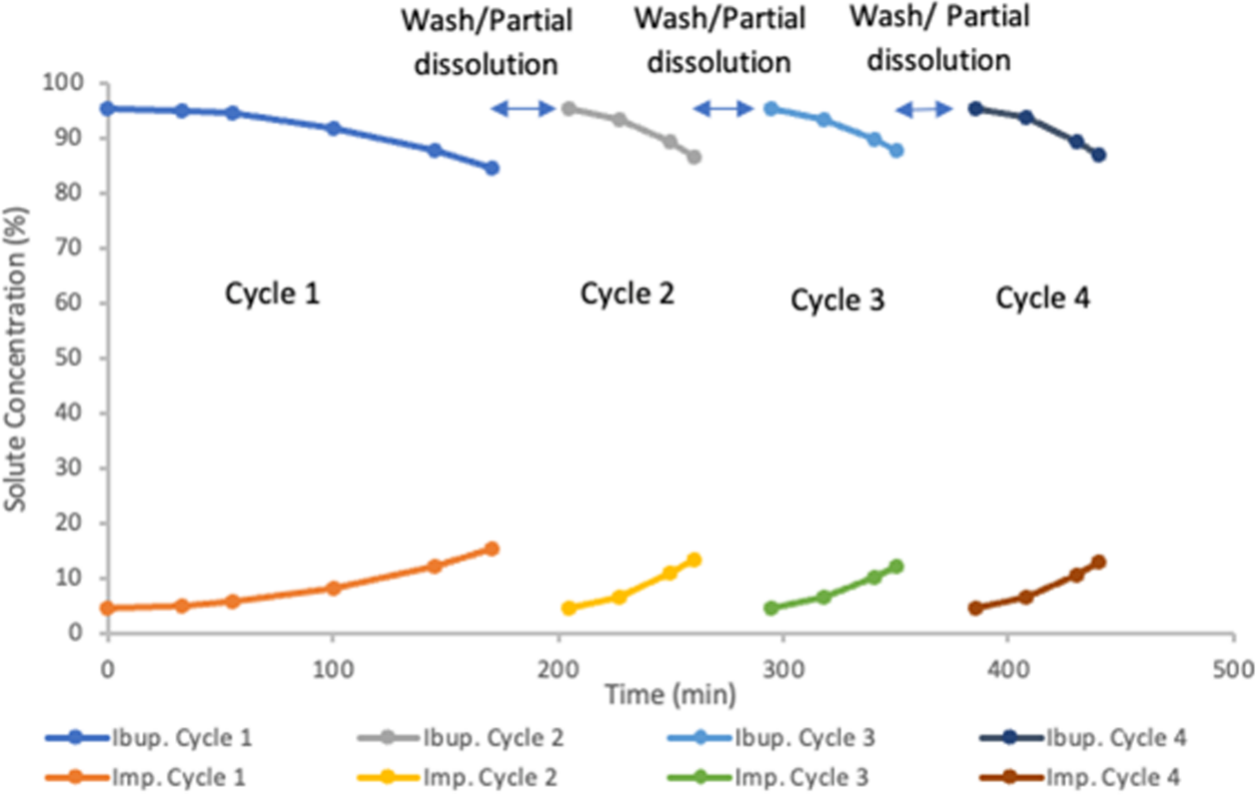
Percentage purity profiles of ibuprofen and impurity in the mother liquor for the cyclical batch experiment using four cycles (by HPLC peak area).

The initial solution for all of the cycles had a purity of 95.33% by the relative peak area of ibuprofen. Samples from the feed solutions were collected during the experiment for each cycle and were analyzed in the HPLC. It was observed that the impurity increased in each cycle, reaching between 12.0 and 15.5% relative peak area, preventing a high accumulation in the mother liquor. A clear, regular pattern for each cycle was observed, where the impurity increased and the ibuprofen decreased until the feed was stopped, followed by the washing and partial dissolution steps. The use of several small feed batches provided a good control of the impurity accumulation on the mother liquor, particularly for cycles 2–4, where the impurities were 13.36, 12.11, and 12.95% by relative peak area, respectively. Figure 4 presents the purity profiles for ibuprofen and impurity in the mother liquor during this experiment with four cycles.

Samples from the dissolved product were analyzed in the HPLC, the ibuprofen purities were 97.90, 98.16, 98.05, and 98.29% by relative peak area for cycles 1–4, respectively. The purity in each cycle was similar to the single-batch experiments conducted previously, meaning that it is possible to achieve a steady operation with controlled impurity concentration in the mother liquor. These results also showed that it was possible to conduct the process for long periods in a cyclical manner without the need to completely stop the process due to accumulation of impurities or the formation of a thick layer of product on the cooling jacket. The dissolved product solutions showed that the yield of this experiment was 76.02% ± 0.52 after the washing step, with a yield of 85.20% ± 0.57 prior to washing.

These results showed that it was possible to conduct many cycles in the FFC, so the induction time for the initial crystal growth could be minimized. The productivity was 0.946 g product/min, considering 335 min of feed time. If two crystallizers are used in parallel to enable the semibatch operation mode crystallization, then it could produce 1.363 kg product/day. Table 1 presents the results for the four-cycle experiment with cyclical batch operation.

## Continuously Fed Operation with Intermittent Partial Removal of Product

**Figure 5:**
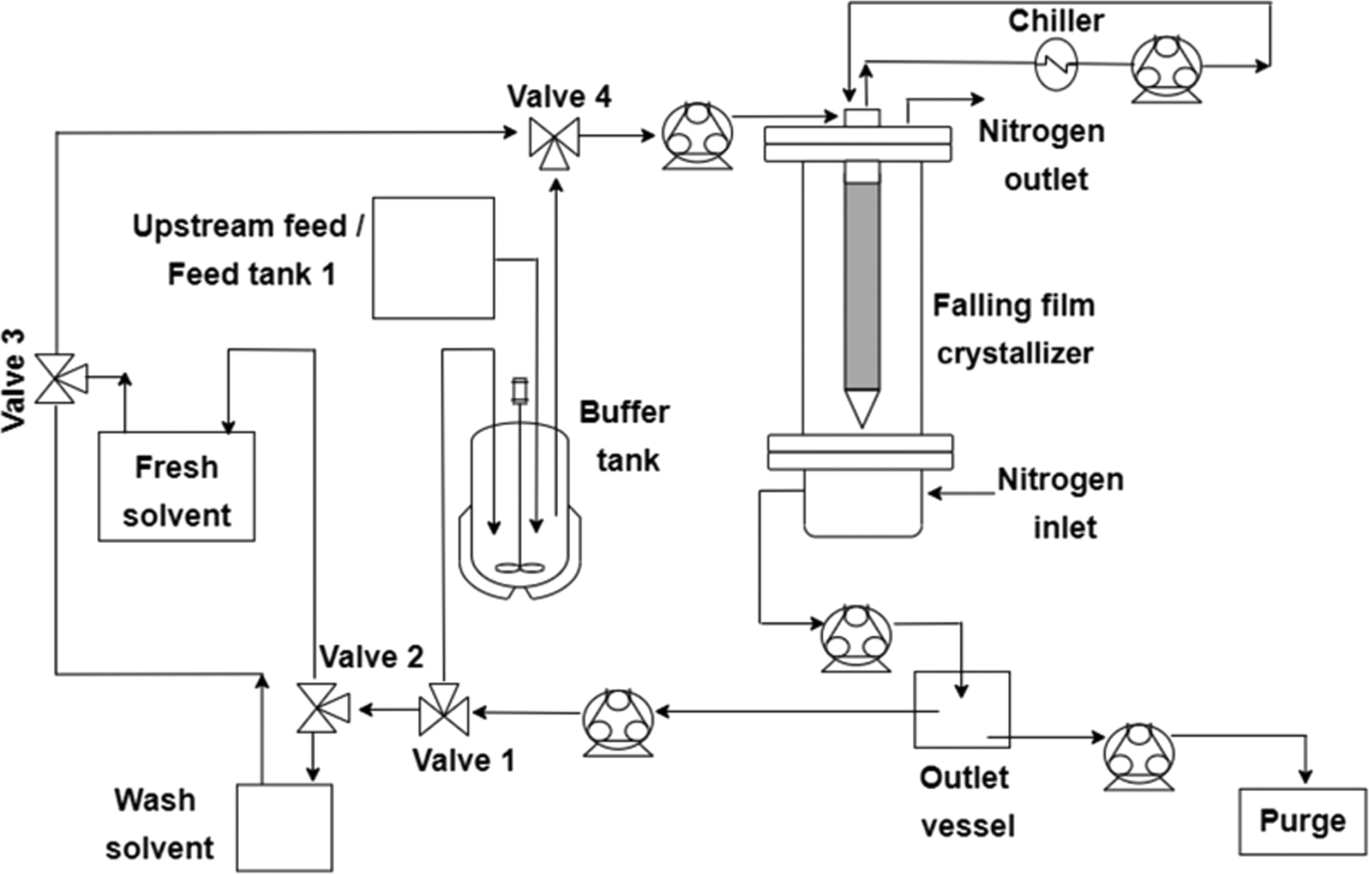
Process diagram for the continuous operation of the FFC.

The option to use continuous feeding directly to the feed buffer tank was also investigated, to simulate an upstream feed from a flow reactor, being captured directly in a single upstream buffer tank from where it undergoes intermediate or final purification. This was achieved by adding an additional feed tank 1 (simulated upstream tank), as per Figure 5, that provided continuous feed into a second feed tank 2 (buffer tank, Figure 5) that operates as per the equivalent feed tank in the single-batch and cyclical batch operation experiments. Additional recovery of product in this purge stream was not conducted within this study, but mother liquor recycle techniques for boosting yield and purity in continuous crystallization will be directly applicable for recycle material from purge stream to feed tank.^43^

The advantage of this operation mode was that the impurities could be kept at a low concentration in combined evaporative and cooling-driven crystallization. In the case of cooling-only-driven solution layer crystallizations, it would allow maintaining supersaturation throughout a given cycle, which will inevitably be depleted in a batch configuration. Cyclical removal of the product with partial or complete dissolution can be used in this operating mode, with partial dissolution again avoiding the low productivity induction time.

**Figure 6:**
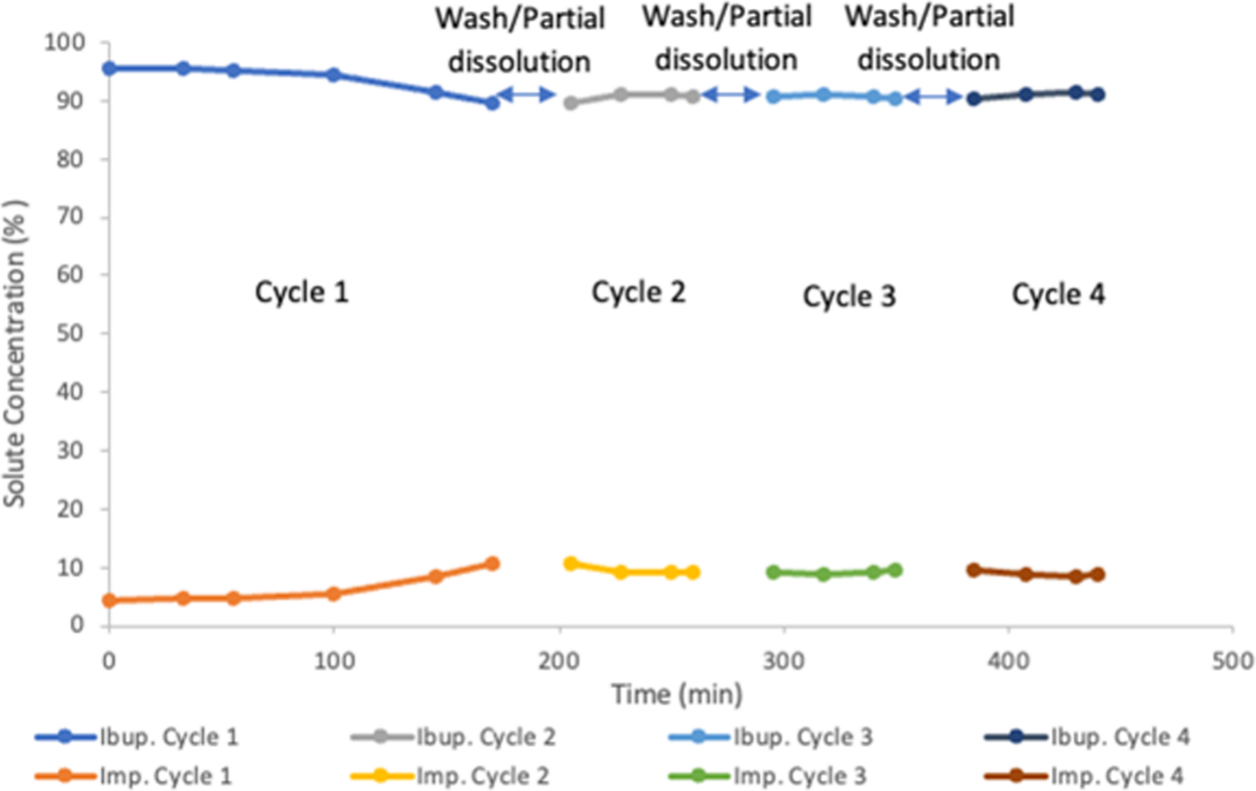
Percentage purity profiles of ibuprofen and impurity in the mother liquor for the continuous feed experiment using four cycles and constant level in feed buffer tank (by HPLC peak area).

The experiment was conducted using four cycles of feed and dissolution of the product. Ibuprofen purity in the initial feed solution was 95.53% by relative peak area (impurity was 4.47% by relative peak area). Samples were taken from the buffer tank during all of the cycles and analyzed in the HPLC to monitor the impurity. Flow rates from the buffer tank, washing step, and dissolution step were kept at 10.50 mL/min in all cycles. For cycle 1, the initial feed solution was 360 mL and the impurity increased with time as in the other experiments because it was conducted without the addition of fresh feed from the simulated upstream tank and without purging. Impurity at time 170 min was 10.53% by relative peak area (at the end of cycle 1). The final volume in the buffer tank was ∼100 mL. Washing was performed with 40 mL of ethanol at 62 °C for 2 min. The partial dissolution was 30 min using 200 mL of ethanol at 62 °C. From cycle 2, the solution from the simulated upstream tank was added to the buffer tank at a flow rate of 2.50–3.0 mL/min and was adjusted manually to maintain a constant volume in the buffer tank. The purge stream was 0.50 mL/min to keep the impurities low in the buffer tank. At the end of cycle 4, all of the ibuprofen was dissolved in 350 mL of ethanol at 62 °C. A decrease in the impurity in the mother liquor was observed, and it was kept roughly constant during the rest of the experiment between 8.5 and 9.5% by relative peak area. Figure 6 presents the ibuprofen and impurity profiles in the mother liquor for this experiment. These results showed that the system could keep the impurity concentration low in the buffer tank for long operation times and that extended stable cyclical operation is viable.

Ibuprofen purities were 97.53, 97.54, 97.05, and 97.53% by relative peak area for cycles 1–4, respectively. The ibuprofen purity was a little lower than in the four-cycle experiment with a washing step using 100 v% ethanol, but the impurity was reduced to around half of the initial concentration in the feed. The yield before the washing step was 76.02% ± 0.43, again lower than the prewash yield 85.20% in the cyclical batch experiments, as would be expected due to the losses in the purge stream. The postwash yield was 67.78% ± 0.36, which was again lower than in the four-cycle batch feed experiment, indicating that while it is efficient to continuously replenish the precrystallizer buffer tank, the use of two batch tanks to feed the process may be preferable. Yield could be increased by reducing the rate of purge flow with an equivalent reduction in feed flow or by further optimizing the wash step. However, the purity specifications will limit the extent to which the purge of material or washing can be reduced. Productivity was found to be 0.90 g ibuprofen/min, which was lower than previous productivity values. Using two units in parallel would produce 1.295 kg product/day. Further optimization of productivity is possible through more rapid evaporation processes or use of antisolvent to increase the operating supersaturation; however, ultimately, the growth kinetics of the system will ultimately set the rate of productivity at maximum.

## Conclusions

A combined, modular, and 3D-printed approach was designed, constructed, and successfully used in a falling film solution layer crystallizer, which incorporated features not compatible with traditional fabrication, such as the highly engineered film distributor. This was readily integrated with modular parts via a printed, threaded section and can be numbered up by hanging multiple-layer crystallization fingers per column. This approach could facilitate more economic deployment of 3D-printed components at larger scales of operation, in this case for implementation of semicontinuous processes in pharmaceuticals for unit operations that do not possess low residence time requirements. The proposed falling film crystallizer showed robust performance in the experiments conducted. The purity of the model API impurity system increased from 95.33 to 98.29% by the relative peak area in cyclical batch operation with a single pass yield of up to 85.20% ± 0.57 before the washing step, indicating an acceptable performance for intermediate purification and solvent swap operations in telescoped flow synthesis. A number of operating modes were presented to increase the productivity of the equipment and demonstrate stable semicontinuous operation. Cyclical batch and continuous feed approaches with partial product dissolution showed an increase in the productivity compared to single-batch operation, due to elimination of the induction time requirements. Operation with direct continuous feed to the process buffer tank with incorporated purge stream was successfully implemented and removed the requirement to switch over between feed tanks.

## Supporting Information Available

Method for the preparation of ibuprofen ethyl ester impurity; further design information relating to the combined modular and 3D-printed falling film crystallizer; brief discussion on printability: limitations and design considerations; and further information of operating modes for falling film crystallization experiments (PDF)
