# Supplementary material for: Design of a Combined Modular and 3D-Printed Falling Film Solution Layer Crystallizer for Intermediate Purification in Continuous Production of Pharmaceuticals
Source: Ind Eng Chem Res. 2021 Jul 12;60(28):10276–85. doi: 10.1021/acs.iecr.1c00988 (PMC8385708; doi:10.1021/acs.iecr.1c00988)
Supplement: Supplementary file 1 — ie1c00988_si_001.pdf [file ie1c00988_si_001.pdf]

**Supporting information for:**

# Design of a combined modular and 3D-printed falling film solution layer crystallizer for intermediate purification in continuous production of pharmaceuticals

Rafael Lopez-Rodriguez, [ab] Matthew J. Harding, [ac] Geoff Gibson, [d] Kevin P. Girard [e], Steven Ferguson [abcf*]

[a] School of Chemical and Bioprocess Engineering, University College Dublin, Belfield, Dublin 4.

[b] SSPC, the SFI Research Centre for Pharmaceuticals, School of Chemical and Bioprocess Engineering, University College Dublin, Belfield, Dublin 4.

[c] I-form, The SFI Research Centre for Advanced Manufacturing, School of Chemical and Bioprocess Engineering, University College Dublin, Belfield, Dublin 4, Ireland

[d] Pfizer Ireland Pharmaceuticals, Ringaskiddy, Ireland

[e] Pfizer Inc. Chemical R&D, Groton, CT, USA 06340

[f] National Institute for Bioprocess Research and Training, 24 Foster's Ave, Belfield, Blackrock, Co. Dublin, A94 X099, Ireland.

* Corresponding authors

1. Preparation of Ibuprofen ethyl ester

20 g of Ibuprofen were put in a 100 ml round bottom flask with a stirrer bar and 40 ml of ethanol were added. Mixture was stirred to fully dissolve the Ibuprofen. 8 drops of sulfuric acid (purity 95.0 – 98.0 %, Sigma Aldrich), adding 2 mL of Ethanol. A condenser was attached to the round bottom flask before start heating in steps up to 90 ˚C. The solution was left stirring overnight. Completion of reaction was checked by TLC using a solution of 10:1

petroleum ether 40 – 60 ˚C (analytical reagent grade, Fisher Scientific) – ethyl acetate (purity > 99.0%, Fisher Scientific).

The product was recovered in a 500 mL separation funnel and mixing it with a solution of 25 ml of ethyl acetate and 25 mL of deionised water. Organic phase was recovered, repeating this step 2 more times. The recovered organic phase was put in the separation funnel with 25 mL of brine. Organic phase was recovered, mixed with an excess of dried magnesium sulphate and stirred by hand. Mixture was filtered to remove the magnesium sulphate. The product was dried in a rotary evaporator. The product was analyzed in the HPLC with purity ~91 % by relative peak area.

2. Design of the Falling film crystallizer

Detailed images of the Falling film crystallizer (FFC) proposed: CAD images and built equipment. Additional details of the bespoke parts used for the construction of the FCC are also provided.

The 3D-printed film distributor has 6 pipes for different connection. Nitrogen outlet is on the side and has an OD of 7.0 mm, ID of 5.0 mm. The rest of the pipes are in the centre of the device. The larger central pipe is the concentric inlet/outlet for the cooling fluid for the heat exchanger. It has an OD of 8.0 mm and ID of 6.0 mm. The inlet of the cooling fluid was an 1/8 inch OD (3.175 mm), ID of 1.57 mm. polyethylene (PE) tubing which was inserted into the film distributor and the heat exchanger, until reaching a height of ~24 mm from the bottom.

Surrounding the cooling pipe, there are 4 pipes which have an OD of 3.75 mm and ID of 1.75 mm. The two taller pipes are for the inlet/outlet of the hot fluid reservoir, which avoids temperature variations in the liquid feed in the open reservoir and prevents crystallization at this part of the equipment. The other two smaller pipes are for inlet of the feed solution to the open reservoir. They are located at opposite sides of the film distributor.

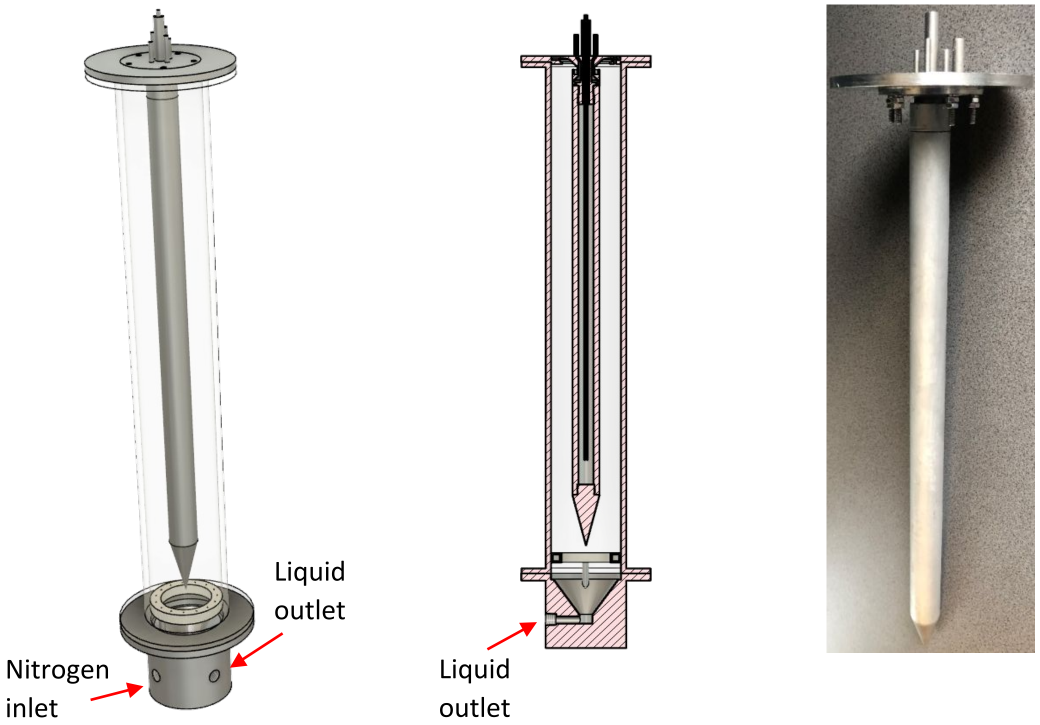


Figure 1. CAD image of the FFC (left), cross-sectional view over the Z axis (center) and film distributor assembled with the flanged section with the cooled heat exchanger and dripping bung (right).

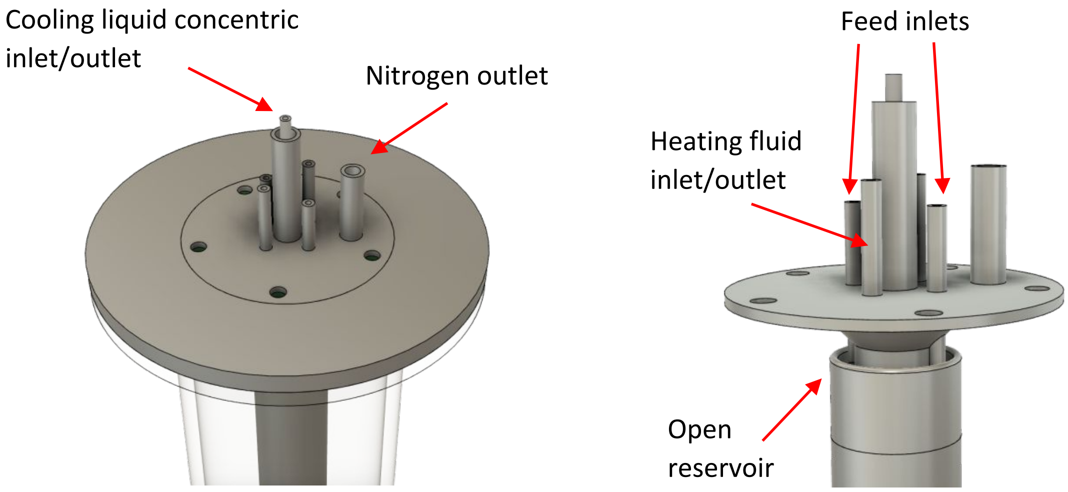


Figure 2. Top view of FFC (left). Film distributor assembled with the cooled heat exchanger (right).

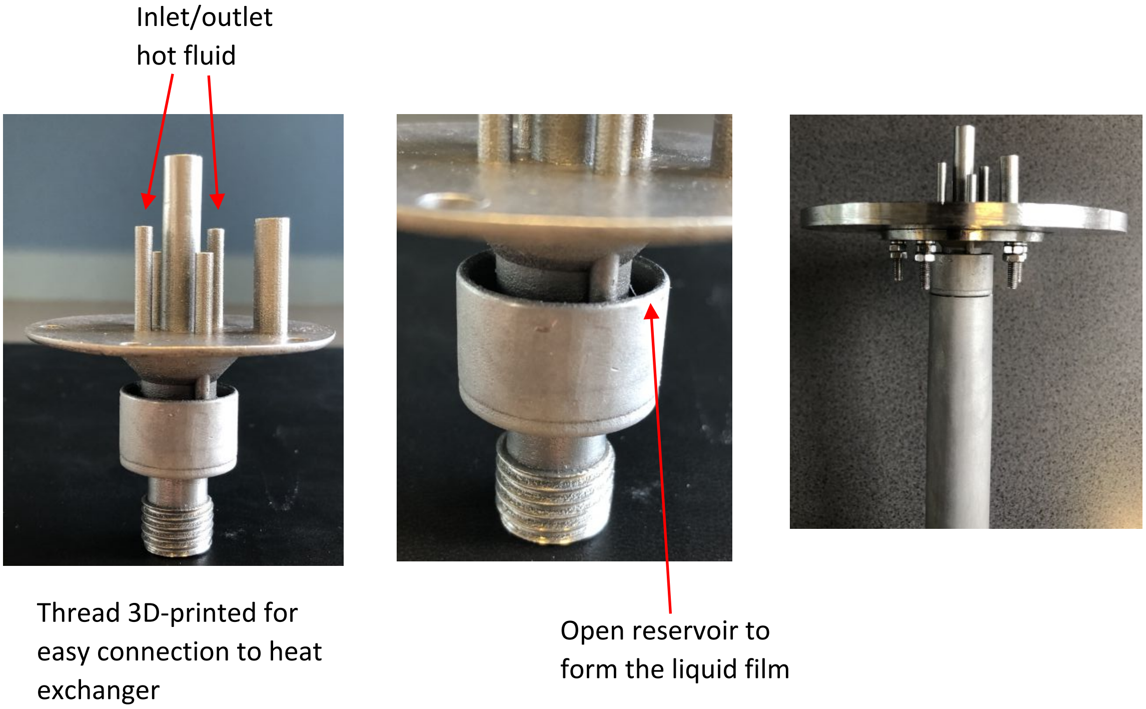

Figure 3. Film distributor 3D-printed in stainless steel (left). Details of the open reservoir for the creation of the liquid film (center). Details of the assembled film distributor with the cooling heat exchanger and the flange (right).

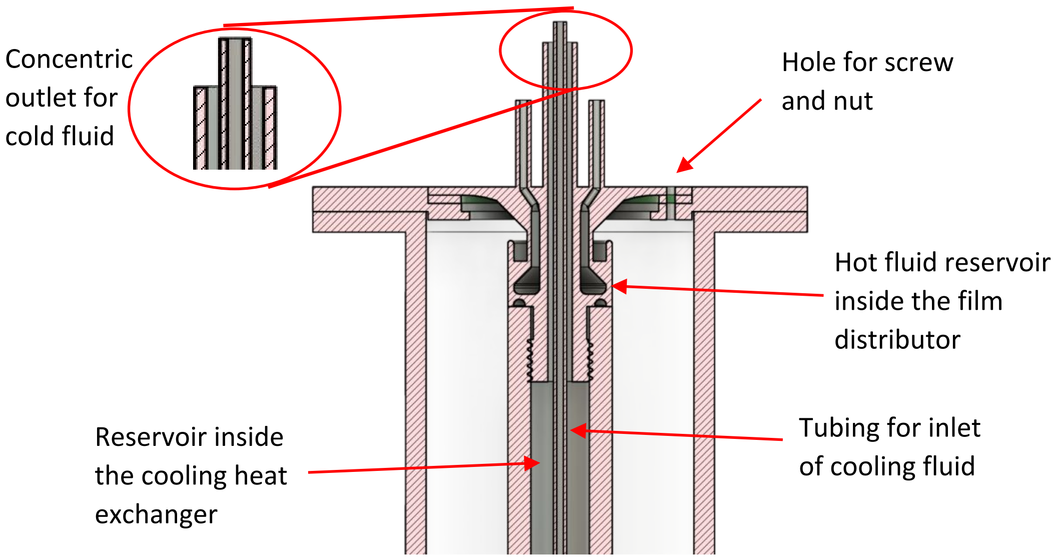


Figure 4. Details of the top part, cross-sectional view over the Z axis.

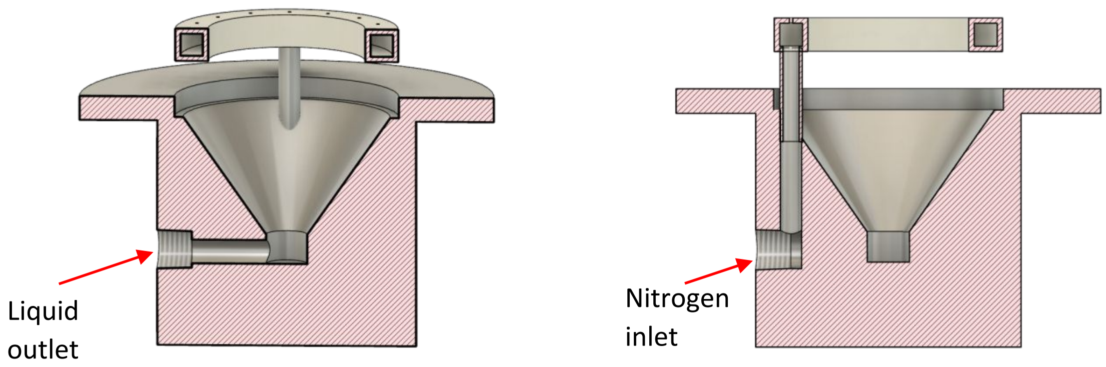

Figure 5. Details of the base, cross-sectional view over the Z axis (left). Details of the base, cross-sectional view over the X axis(right).

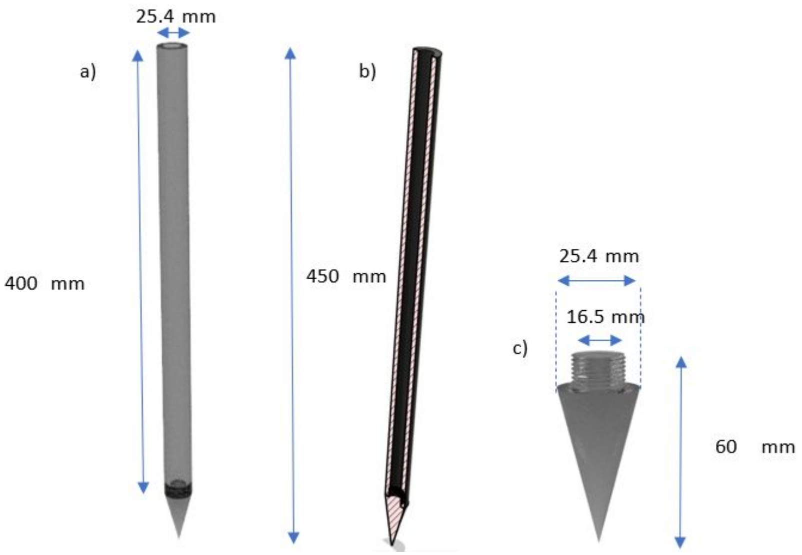


Figure 6. a) Details of the cooled heat exchanger, b) Cross sectional view of the cooling (centre) and c) Details of the dripping bung.

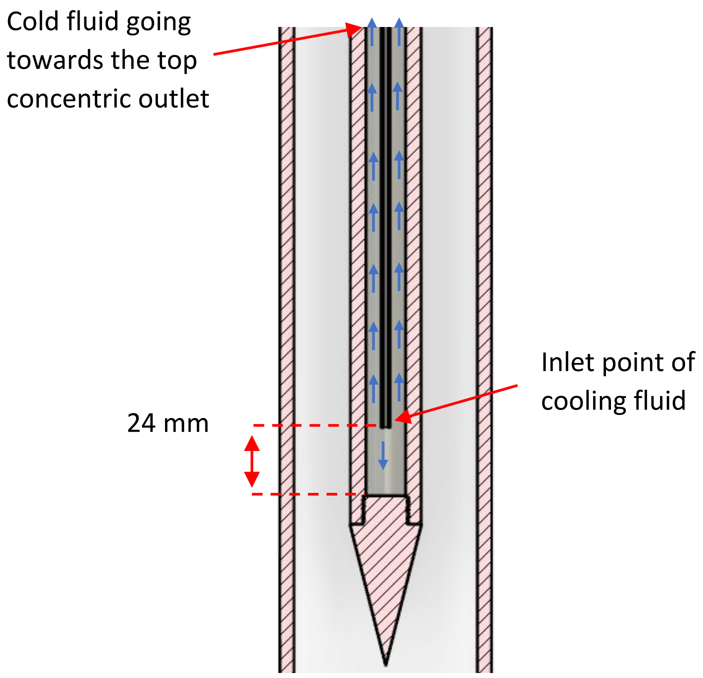

Figure 7. Details of cooling fluid inlet point inside the heat exchanger, cross-sectional view over the X axis.

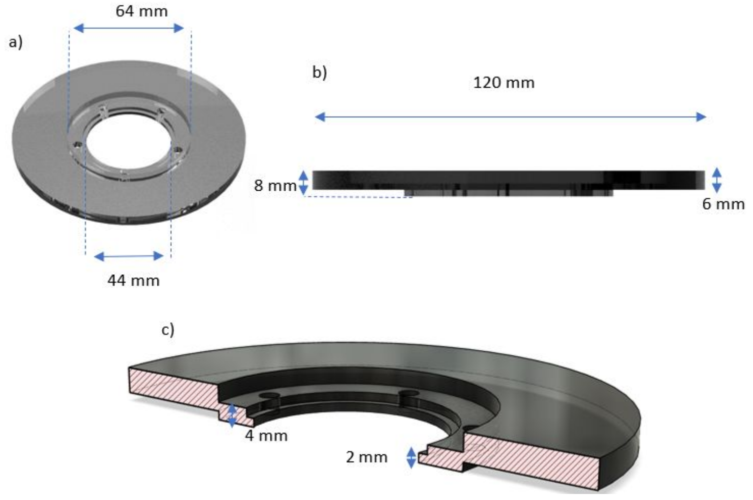


Figure 8. Details of the top bespoke flange built for the Falling Film Solution Layer Crystallizer: a) Isometric view, b) Frontal view and c) Cross sectional view. Holes in the flange have a diameter of 4.75 mm.

## 3. Printability: limitations and design considerations

It is important that the device be compatible with the printed methodology to be utilized, in this case selective laser melting based printing of 316L stainless steel. While 3D-printing provides significant benefits in fabricating components with internal structures in a single part, limitations exist particularly with respect to the inclusion of overhanging faces within the design. As such, overhanging faces were avoided, and where they could not be, arched

designs were implemented, for example within the hot fluid reservoir the arched ceilings with 45 degree angles were utilized in place of a flat 90 degree overhang in ceiling, tapered walls were employed to narrow the diameter of this chamber from the top to the bottom of the device. In addition, no completely enclosed chambers were utilized, relatively large bore inlets and outlets led in and out of the internal reservoir enabled clearing of residual metal powders from the print. That said the critical parts such as the trough and distributor were external, with the internal sections relatively open and therefore able avoid the powder clearing postprocessing problems that can be prohibitive in printing long narrow channels often associated with flow chemistry devices in SLM.

Another limitation is scale, while the scale and speed of additive manufacturing is increasing, large scale prints can be expensive to conduct. In the printer utilized for this study, the entire working section for the crystallizer could not be printed in one part. The modular nature could facilitate printing multiple pipe segments with alternate male and female thread sections allowing for a long segment in excess of the maximum build dimension to be put together if desired. In general, it is unlikely to be significantly beneficial to print standard geometries such as pipes or tubes. However, if needed, modular tubular sections with engineered surfaces to improve heat transfer during layer crystallization and facilitate structural support could be

incorporated. This has previously been shown to allow intensification of a falling film melt crystallization where smoothed surface, helicoidal micro-grooves and stainless steel fins welded into the cooling surface were investigated for the production of acrylic acid.[42]

4. Operation of the Falling film crystallizer

For single batch experiment, it was used 218.37 g of Ibuprofen, 10.92 g of Ibuprofen ethyl ester, 87.30 g ethanol and 21.89 g of deionized water. The feed solution resulting was ~375 mL when the Ibuprofen was dissolved. The feed solution was recirculated for 200 minutes to allow the growth of the crystal layer and the flow rate used was 10.50 mL/min. The feed was evaporated and ibuprofen was crystallized until 90~100 mL of the solution was left, including the solution recirculating in the pipelines. This would allow the recovery product with purity above 98% by relative peak area. Lower volumes could be reached in the feed vessel, but the impurity accumulates in the mother liquor and could cause a lower purity of the product. 100 v% ethanol preheated in the water bath at 62 °C was used as the washing step to remove any mother liquor remaining in the pipes, the film distributor and in the crystal layer. It also removed the impurities that could be trapped in the outer part of the crystal layer. The washing step was conducted for 2 min at 10.50 mL/min, recirculating the liquid into the designated bottle. The crystal layer was dissolved completely using 350 mL of 100 v% ethanol preheated at 62 °C, with a flow rate of 10.50 mL/min and recirculating into the designated bottle, usually taking ~90 minutes to complete this step. A final washing step with 100 v% ethanol for 2 minutes at 10.50 mL/min was conducted to remove any product left in the FFC or in the pipes. Figure 9 presents a process schematic for the single batch operational

mode. In the experiment, the feed tank was replaced by the washing bottles (250 mL Duran bottles) and dissolved product bottles (500 mL Duran bottles) as required.

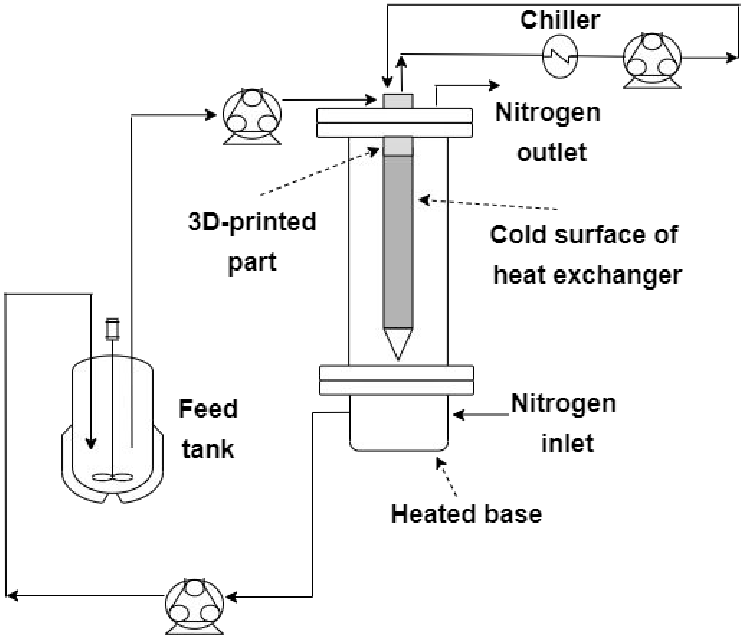


Figure 9. Process diagram for the single batch operation of the FFC.

For the cyclical batch experiments, the experiments were conducted using 4 cycles in total to demonstrate that the process could work indefinitely using this operation mode. Additionally, the induction period of approximately 60 minutes for nucleation and growth of the initial crystal layer had the slowest rate of mass deposition and hence productivity. From this initial period, the first crystals were observed typically at 10 minutes of operation and more crystals appearing until most of the cooling jacket was covered at 60 minutes. To improve the

productivity, the batch feed runs were conducted as several cycles performed in quick succession with partial dissolution, rather than complete dissolution between the cycles, eliminating this less productive induction period. Process conditions were kept the same as in single batch experiment for comparisons. The feed used in this experiment was equivalent to 2 batches, which was divided in different bottles for each cycle to get a better control of the process. The first cycle was used to grow the initial layer of the crystals so 375 mL were used as the initial feed (in a 1.0 L Duran bottle), which was equivalent to the volume for 1 single batch experiment. Cycles 2, 3 and 4 were conducted using 125 mL each, which represented 1/3 of the volume of a single batch each (in 250 mL Duran bottles). Flow rate was kept at 10.50 ml/min for feed solutions, washing steps and dissolution steps. Feed 1 was recirculated for 170 minutes, then the pipes were rinsed with 40 mL of 100 v% Ethanol preheated at 62 °C for 2 minutes (in a 250 mL Duran bottle), 200 mL of 100 v% Ethanol (using a 500 mL Duran bottle) preheated at 62 °C was added to dissolve part of the crystal bed and was recirculated for 30 minutes. The feed solution from the next cycle was circulated on top of the residual crystal layer, which eliminated the induction time. This cycle of partial dissolution and crystal growth could be achieved several times. For cycle 2, feed from the feed 2 bottle was pumped into the crystallizer and the solution was recirculated for 55

minutes, then the pipes were rinsed with 100 v% ethanol for 2 minutes and the product was partially dissolved in 100 v% ethanol for 30 minutes, as per cycle 1. Cycles 3 and 4 were conducted in as per cycle 2. For the last cycle, a complete dissolution of the crystal layer could be conducted to prevent unnecessary loss in yield. At the end of cycle 4, all the crystals were fully re-dissolved in 350 mL of 100 v% ethanol preheated at 62 °C and a final washing step was conducted to clear process lines.

For continuously fed experiments with intermittent removal of product, a continuous upstream feed could be collected in a feed tank (Feed tank 1/ upstream feed tank, Figure 10), which subsequently would feed a secondary feed tank (Buffer tank, Figure 10), to simulate feed flow from a continuous upstream operation. Solution in buffer tank (1.0 L Duran bottle) would feed the FFC as in previous operation modes. An auxiliary outlet vessel was added to collect the outlet stream (250 mL Duran bottle), where a purge flow rate was set to 0.5 mL/min (~5 % of the feed flow rate) to control the impurity concentration in the buffer tank. The remaining solution from this vessel was recycled back into the buffer tank. The fresh feed from the simulated upstream tank would replace the evaporated solvent, the crystallized API on the FFC and the solution lost in the purge stream. Buffer tank operated at constant level which secured a low impurity concentration and maintained supersaturation in the

mother liquor. If the impurity concentration is kept constant and low for extended periods, one of the primary reasons for stopping the process is removed. The total feed volume was 2.5 times the volume of a single batch experiment to compensate the solution lost in the purge stream. Cycle 1 was carried out using 360 mL of the feed, with a flow rate of 10.50 mL/min and was used to grow the initial crystal layer as in the other experiments, so there was no addition from the simulated upstream tank to the buffer thank and no purge stream. After 170 minutes, the feed was stopped and 100 v% ethanol was pumped for 2 minutes as the washing step at 10.50 mL/min. The product was partially dissolved using 200 mL of ethanol and recycling the solution for 30 minutes at flow rate of 10.50mL/min. At the end of cycle 1, the remaining solution was ~100 mL in the buffer tank which was tried to be kept constant from Cycle 2. The remaining feed solution (560 mL) was kept in the simulated upstream tank. For cycles 2, 3 and 4, the initial feed from the simulated upstream tank was pumped at a flow rate between 2.5 – 3.0 mL/min into the buffer tank, adjusting the flow rate manually if the liquid level changed significantly. This feed solution was pumped into the FFC for 55 minutes, followed by washing for 2 minutes with 100 v% ethanol preheated at 62 ˚C for cycles 2 to 4. Partial dissolution of the product with 100 v% ethanol preheated at 62 ˚C

for 30 min was conducted at the end of cycles 2 and 3 and complete dissolution of the crystal layer with 100 v% ethanol at the end of cycle 4.

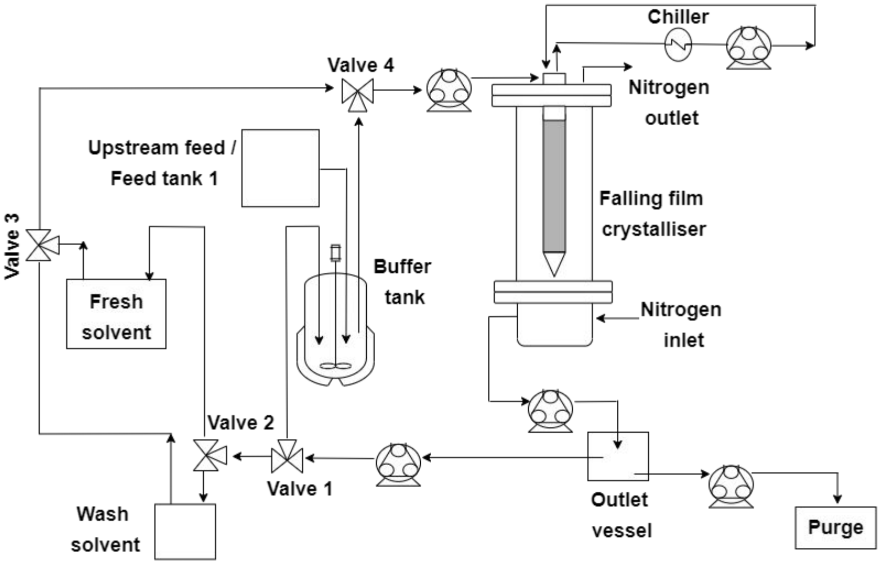


Figure 10. Process diagram for the continuous operation of the FFC.
